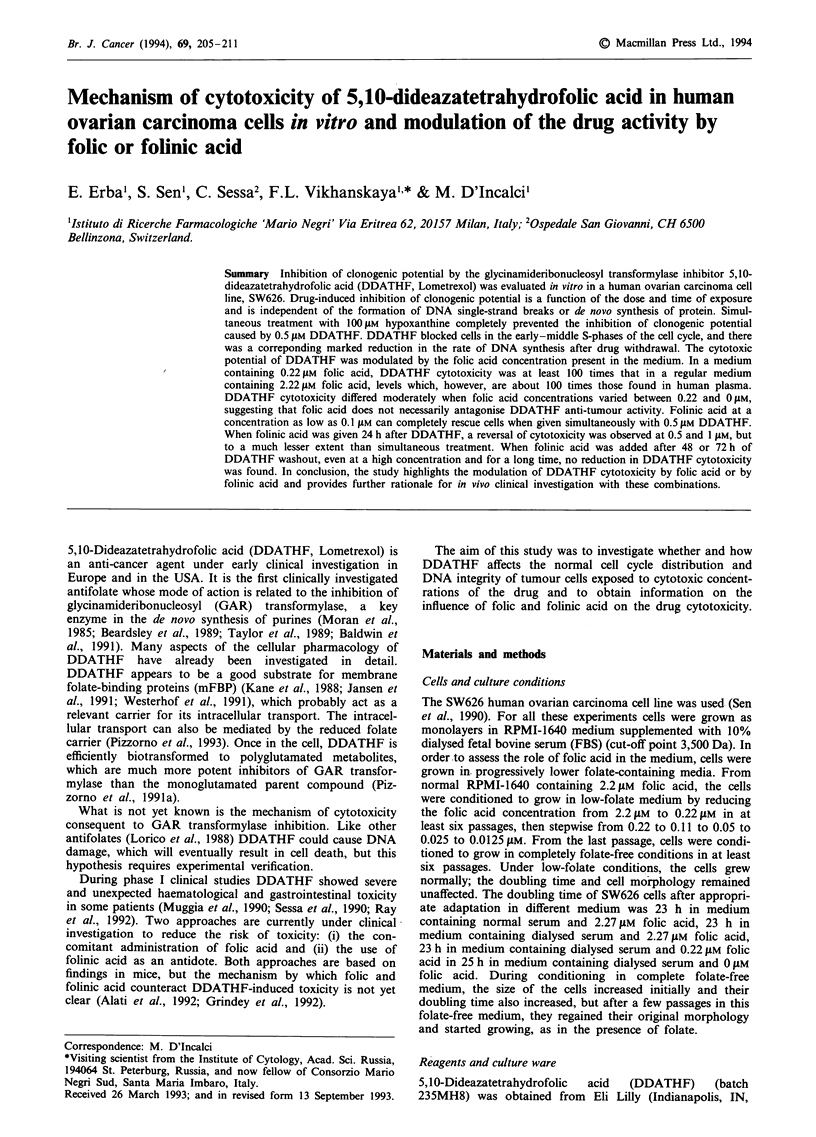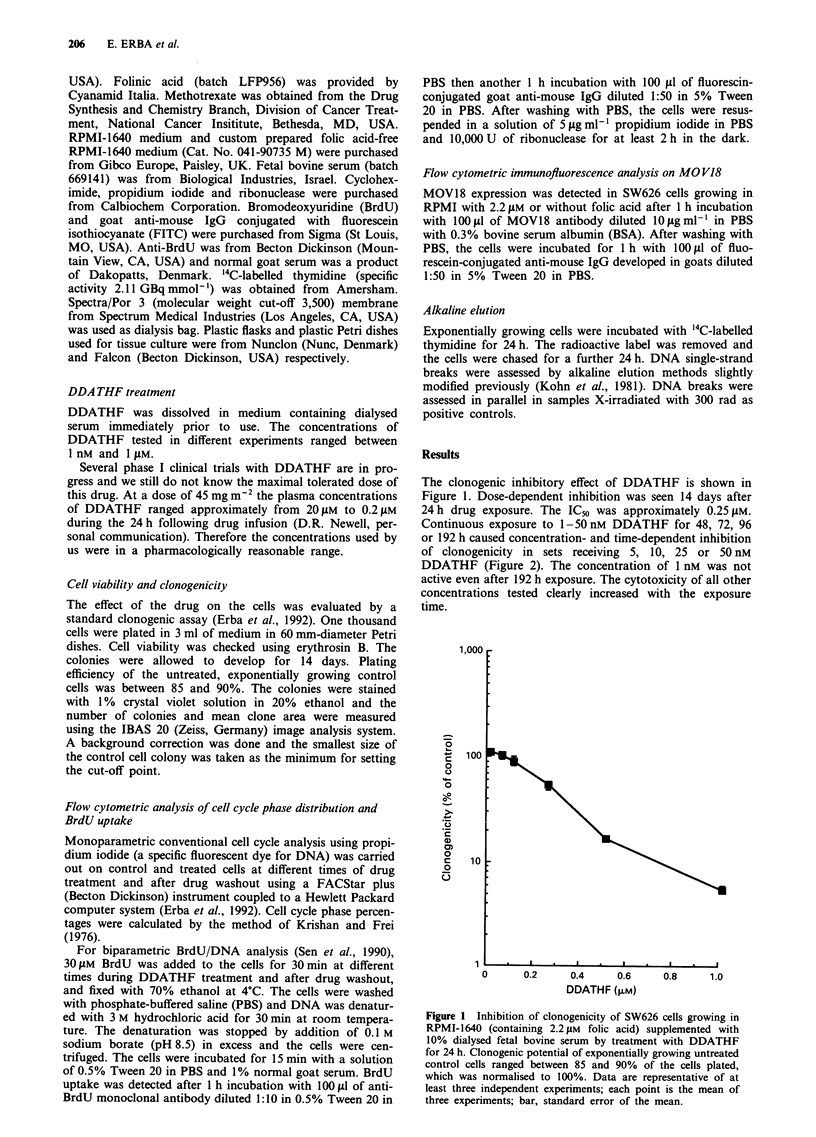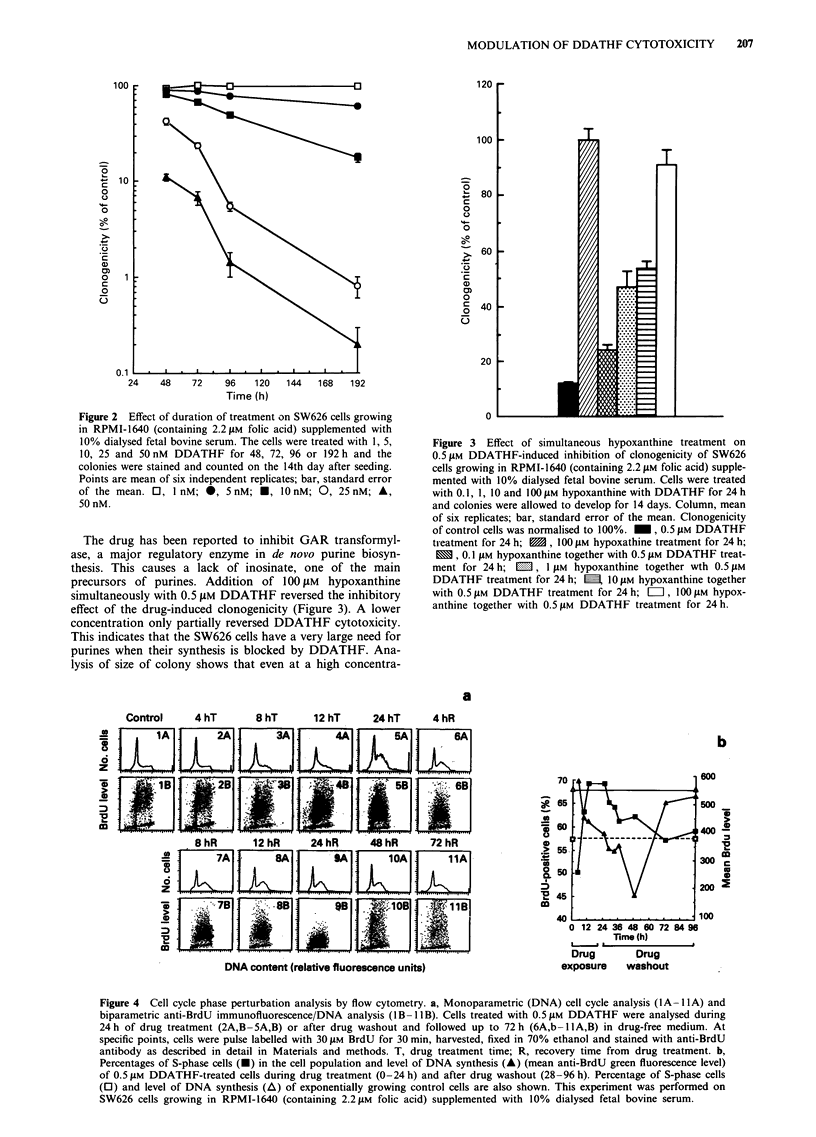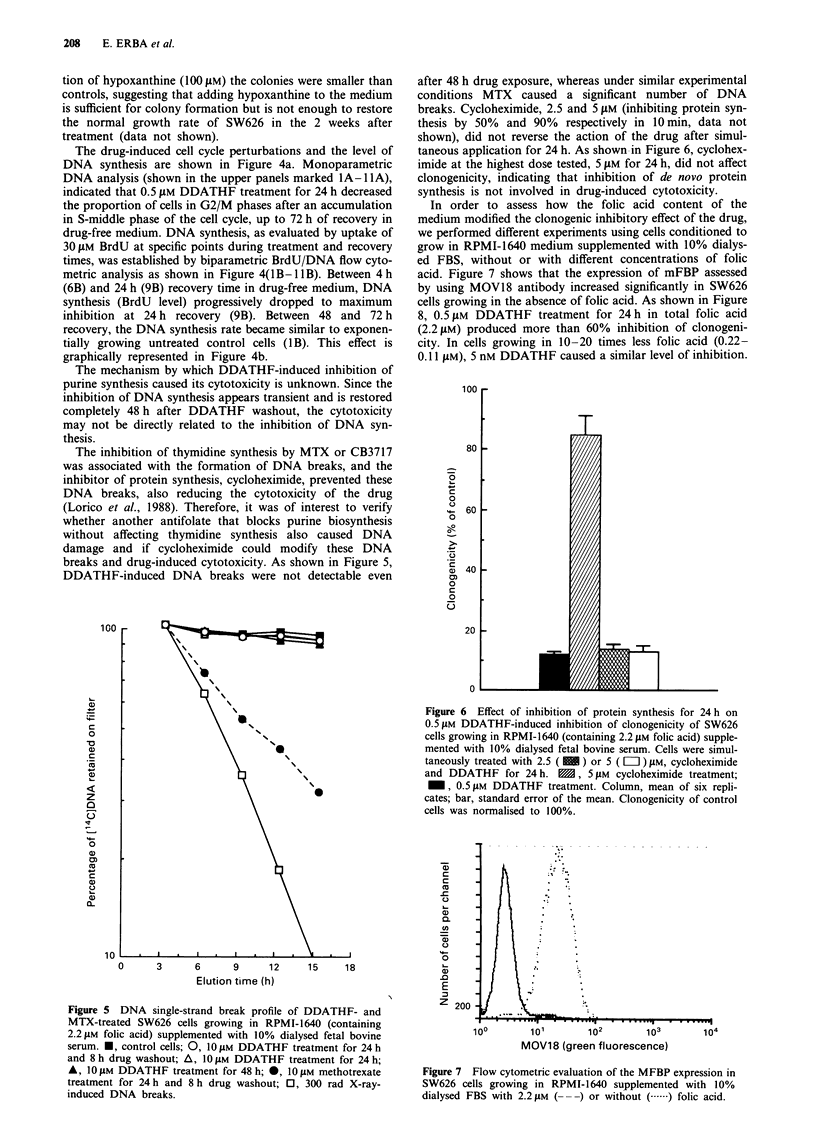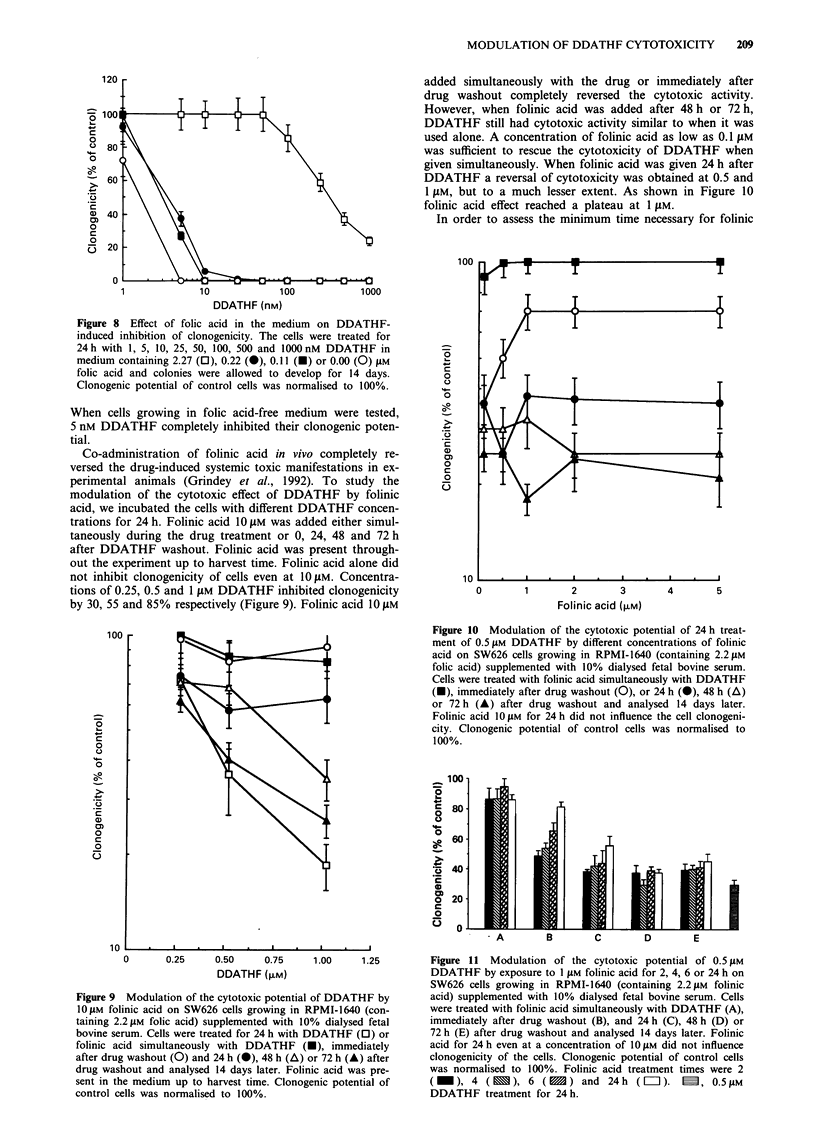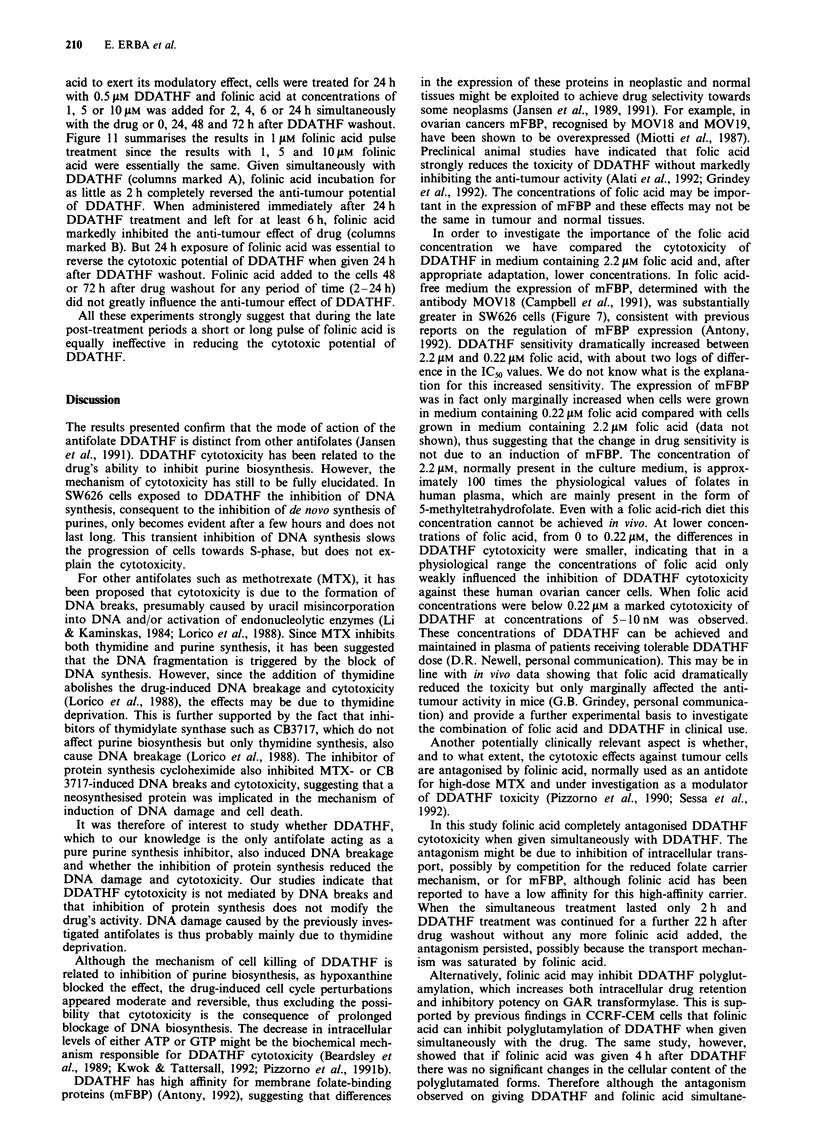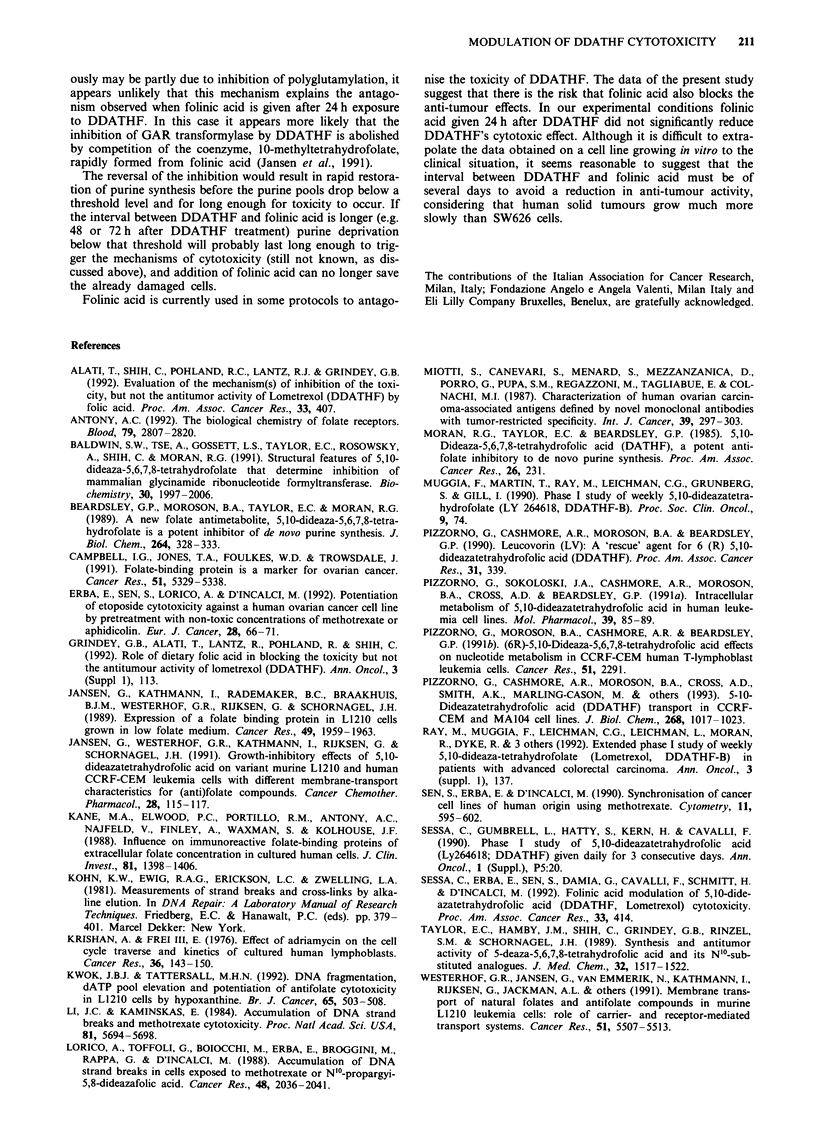# Mechanism of cytotoxicity of 5,10-dideazatetrahydrofolic acid in human ovarian carcinoma cells in vitro and modulation of the drug activity by folic or folinic acid.

**DOI:** 10.1038/bjc.1994.40

**Published:** 1994-02

**Authors:** E. Erba, S. Sen, C. Sessa, F. L. Vikhanskaya, M. D'Incalci

**Affiliations:** Istituto di Ricerche Farmacologiche Mario Negri, Milan, Italy.

## Abstract

Inhibition of clonogenic potential by the glycinamideribonucleosyl transformylase inhibitor 5,10-dideazatetrahydrofolic acid (DDATHF, Lometrexol) was evaluated in vitro in a human ovarian carcinoma cell line, SW626. Drug-induced inhibition of clonogenic potential is a function of the dose and time of exposure and is independent of the formation of DNA single-strand breaks or de novo synthesis of protein. Simultaneous treatment with 100 microM hypoxanthine completely prevented the inhibition of clonogenic potential caused by 0.5 microM DDATHF. DDATHF blocked cells in the early-middle S-phases of the cell cycle, and there was a corresponding marked reduction in the rate of DNA synthesis after drug withdrawal. The cytotoxic potential of DDATHF was modulated by the folic acid concentration present in the medium. In a medium containing 0.22 microM folic acid, DDATHF cytotoxicity was at least 100 times that in a regular medium containing 2.22 microM folic acid, levels which, however, are about 100 times those found in human plasma. DDATHF cytotoxicity differed moderately when folic acid concentrations varied between 0.22 and 0 microM, suggesting that folic acid does not necessarily antagonise DDATHF anti-tumour activity. Folinic acid at a concentration as low as 0.1 microM can completely rescue cells when given simultaneously with 0.5 microM DDATHF. When folinic acid was given 24 h after DDATHF, a reversal of cytotoxicity was observed at 0.5 and 1 microM, but to a much lesser extent than simultaneous treatment. When folinic acid was added after 48 or 72 h of DDATHF washout, even at a high concentration and for a long time, no reduction in DDATHF cytotoxicity was found. In conclusion, the study highlights the modulation of DDATHF cytotoxicity by folic acid or by folinic acid and provides further rationale for in vivo clinical investigation with these combinations.


*Br. J. Cancer* (1994), **69**, 205–211 © Macmillan Press Ltd., 1994

### Mechanism of cytotoxicity of 5,10-dideazatetrahydrofolic acid in human ovarian carcinoma cells *in vitro* and modulation of the drug activity by folic or folinic acid

E. Erba[1], S. Sen[1], C. Sessa[2], F.L. Vikhanskaya[1,]* & M. D'Incalci[1]

[1]*Istituto di Ricerche Farmacologiche 'Mario Negri' Via Eritrea 62, 20157 Milan, Italy;* [2]*Ospedale San Giovanni, CH 6500 Bellinzona, Switzerland.*

**Summary** Inhibition of clonogenic potential by the glycinamideribonucleosyl transformylase inhibitor 5,10-dideazatetrahydrofolic acid (DDATHF, Lometrexol) was evaluated *in vitro* in a human ovarian carcinoma cell line, SW626. Drug-induced inhibition of clonogenic potential is a function of the dose and time of exposure and is independent of the formation of DNA single-strand breaks or *de novo* synthesis of protein. Simultaneous treatment with 100 μM hypoxanthine completely prevented the inhibition of clonogenic potential caused by 0.5 μM DDATHF. DDATHF blocked cells in the early–middle S-phases of the cell cycle, and there was a correponding marked reduction in the rate of DNA synthesis after drug withdrawal. The cytotoxic potential of DDATHF was modulated by the folic acid concentration present in the medium. In a medium containing 0.22 μM folic acid, DDATHF cytotoxicity was at least 100 times that in a regular medium containing 2.22 μM folic acid, levels which, however, are about 100 times those found in human plasma. DDATHF cytotoxicity differed moderately when folic acid concentrations varied between 0.22 and 0 μM, suggesting that folic acid does not necessarily antagonise DDATHF anti-tumour activity. Folinic acid at a concentration as low as 0.1 μM can completely rescue cells when given simultaneously with 0.5 μM DDATHF. When folinic acid was given 24 h after DDATHF, a reversal of cytotoxicity was observed at 0.5 and 1 μM, but to a much lesser extent than simultaneous treatment. When folinic acid was added after 48 or 72 h of DDATHF washout, even at a high concentration and for a long time, no reduction in DDATHF cytotoxicity was found. In conclusion, the study highlights the modulation of DDATHF cytotoxicity by folic acid or by folinic acid and provides further rationale for *in vivo* clinical investigation with these combinations.

5,10-Dideazatetrahydrofolic acid (DDATHF, Lometrexol) is an anti-cancer agent under early clinical investigation in Europe and in the USA. It is the first clinically investigated antifolate whose mode of action is related to the inhibition of glycinamideribonucleosyl (GAR) transformylase, a key enzyme in the *de novo* synthesis of purines (Moran *et al.*, 1985; Beardsley *et al.*, 1989; Taylor *et al.*, 1989; Baldwin *et al.*, 1991). Many aspects of the cellular pharmacology of DDATHF have already been investigated in detail. DDATHF appears to be a good substrate for membrane folate-binding proteins (mFBP) (Kane *et al.*, 1988; Jansen *et al.*, 1991; Westerhof *et al.*, 1991), which probably act as a relevant carrier for its intracellular transport. The intracellular transport can also be mediated by the reduced folate carrier (Pizzorno *et al.*, 1993). Once in the cell, DDATHF is efficiently biotransformed to polyglutamated metabolites, which are much more potent inhibitors of GAR transformylase than the monoglutamated parent compound (Pizzorno *et al.*, 1991a).

What is not yet known is the mechanism of cytotoxicity consequent to GAR transformylase inhibition. Like other antifolates (Lorico *et al.*, 1988) DDATHF could cause DNA damage, which will eventually result in cell death, but this hypothesis requires experimental verification.

During phase I clinical studies DDATHF showed severe and unexpected haematological and gastrointestinal toxicity in some patients (Muggia *et al.*, 1990; Sessa *et al.*, 1990; Ray *et al.*, 1992). Two approaches are currently under clinical investigation to reduce the risk of toxicity: (i) the concomitant administration of folic acid and (ii) the use of folinic acid as an antidote. Both approaches are based on findings in mice, but the mechanism by which folic and folinic acid counteract DDATHF-induced toxicity is not yet clear (Alati *et al.*, 1992; Grindey *et al.*, 1992).

Correspondence: M. D'Incalci
*Visiting scientist from the Institute of Cytology, Acad. Sci. Russia, 194064 St. Peterburg, Russia, and now fellow of Consorzio Mario Negri Sud, Santa Maria Imbaro, Italy.
Received 26 March 1993; and in revised form 13 September 1993.

The aim of this study was to investigate whether and how DDATHF affects the normal cell cycle distribution and DNA integrity of tumour cells exposed to cytotoxic concentrations of the drug and to obtain information on the influence of folic and folinic acid on the drug cytotoxicity.

### Materials and methods

### *Cells and culture conditions*

The SW626 human ovarian carcinoma cell line was used (Sen *et al.*, 1990). For all these experiments cells were grown as monolayers in RPMI-1640 medium supplemented with 10% dialysed fetal bovine serum (FBS) (cut-off point 3,500 Da). In order to assess the role of folic acid in the medium, cells were grown in progressively lower folate-containing media. From normal RPMI-1640 containing 2.2 μM folic acid, the cells were conditioned to grow in low-folate medium by reducing the folic acid concentration from 2.2 μM to 0.22 μM in at least six passages, then stepwise from 0.22 to 0.11 to 0.05 to 0.025 to 0.0125 μM. From the last passage, cells were conditioned to grow in completely folate-free conditions in at least six passages. Under low-folate conditions, the cells grew normally; the doubling time and cell morphology remained unaffected. The doubling time of SW626 cells after appropriate adaptation in different medium was 23 h in medium containing normal serum and 2.27 μM folic acid, 23 h in medium containing dialysed serum and 2.27 μM folic acid, 23 h in medium containing dialysed serum and 0.22 μM folic acid in 25 h in medium containing dialysed serum and 0 μM folic acid. During conditioning in complete folate-free medium, the size of the cells increased initially and their doubling time also increased, but after a few passages in this folate-free medium, they regained their original morphology and started growing, as in the presence of folate.

### *Reagents and culture ware*

5,10-Dideazatetrahydrofolic acid (DDATHF) (batch 235MH8) was obtained from Eli Lilly (Indianapolis, IN,

USA). Folinic acid (batch LFP956) was provided by Cyanamid Italia. Methotrexate was obtained from the Drug Synthesis and Chemistry Branch, Division of Cancer Treatment, National Cancer Insititute, Bethesda, MD, USA. RPMI-1640 medium and custom prepared folic acid-free RPMI-1640 medium (Cat. No. 041-90735 M) were purchased from Gibco Europe, Paisley, UK. Fetal bovine serum (batch 669141) was from Biological Industries, Israel. Cycloheximide, propidium iodide and ribonuclease were purchased from Calbiochem Corporation. Bromodeoxyuridine (BrdU) and goat anti-mouse IgG conjugated with fluorescein isothiocyanate (FITC) were purchased from Sigma (St Louis, MO, USA). Anti-BrdU was from Becton Dickinson (Mountain View, CA, USA) and normal goat serum was a product of Dakopatts, Denmark. $^{14}$C-labelled thymidine (specific activity 2.11 GBq mmol$^{-1}$) was obtained from Amersham. Spectra/Por 3 (molecular weight cut-off 3,500) membrane from Spectrum Medical Industries (Los Angeles, CA, USA) was used as dialysis bag. Plastic flasks and plastic Petri dishes used for tissue culture were from Nunclon (Nunc, Denmark) and Falcon (Becton Dickinson, USA) respectively.

### *DDATHF treatment*

DDATHF was dissolved in medium containing dialysed serum immediately prior to use. The concentrations of DDATHF tested in different experiments ranged between 1 nM and 1 $\mu$M.

Several phase I clinical trials with DDATHF are in progress and we still do not know the maximal tolerated dose of this drug. At a dose of 45 mg m$^{-2}$ the plasma concentrations of DDATHF ranged approximately from 20 $\mu$M to 0.2 $\mu$M during the 24 h following drug infusion (D.R. Newell, personal communication). Therefore the concentrations used by us were in a pharmacologically reasonable range.

### *Cell viability and clonogenicity*

The effect of the drug on the cells was evaluated by a standard clonogenic assay (Erba et al., 1992). One thousand cells were plated in 3 ml of medium in 60 mm-diameter Petri dishes. Cell viability was checked using erythrosin B. The colonies were allowed to develop for 14 days. Plating efficiency of the untreated, exponentially growing control cells was between 85 and 90%. The colonies were stained with 1% crystal violet solution in 20% ethanol and the number of colonies and mean clone area were measured using the IBAS 20 (Zeiss, Germany) image analysis system. A background correction was done and the smallest size of the control cell colony was taken as the minimum for setting the cut-off point.

### *Flow cytometric analysis of cell cycle phase distribution and BrdU uptake*

Monoparametric conventional cell cycle analysis using propidium iodide (a specific fluorescent dye for DNA) was carried out on control and treated cells at different times of drug treatment and after drug washout using a FACStar plus (Becton Dickinson) instrument coupled to a Hewlett Packard computer system (Erba et al., 1992). Cell cycle phase percentages were calculated by the method of Krishan and Frei (1976).

For biparametric BrdU/DNA analysis (Sen et al., 1990), 30 $\mu$M BrdU was added to the cells for 30 min at different times during DDATHF treatment and after drug washout, and fixed with 70% ethanol at 4°C. The cells were washed with phosphate-buffered saline (PBS) and DNA was denatured with 3 M hydrochloric acid for 30 min at room temperature. The denaturation was stopped by addition of 0.1 M sodium borate (pH 8.5) in excess and the cells were centrifuged. The cells were incubated for 15 min with a solution of 0.5% Tween 20 in PBS and 1% normal goat serum. BrdU uptake was detected after 1 h incubation with 100 $\mu$l of anti-BrdU monoclonal antibody diluted 1:10 in 0.5% Tween 20 in PBS then another 1 h incubation with 100 $\mu$l of fluorescin-conjugated goat anti-mouse IgG diluted 1:50 in 5% Tween 20 in PBS. After washing with PBS, the cells were resuspended in a solution of 5 $\mu$g ml$^{-1}$ propidium iodide in PBS and 10,000 U of ribonuclease for at least 2 h in the dark.

### *Flow cytometric immunofluorescence analysis on MOV18*

MOV18 expression was detected in SW626 cells growing in RPMI with 2.2 $\mu$M or without folic acid after 1 h incubation with 100 $\mu$l of MOV18 antibody diluted 10 $\mu$g ml$^{-1}$ in PBS with 0.3% bovine serum albumin (BSA). After washing with PBS, the cells were incubated for 1 h with 100 $\mu$l of fluorescein-conjugated anti-mouse IgG developed in goats diluted 1:50 in 5% Tween 20 in PBS.

### *Alkaline elution*

Exponentially growing cells were incubated with $^{14}$C-labelled thymidine for 24 h. The radioactive label was removed and the cells were chased for a further 24 h. DNA single-strand breaks were assessed by alkaline elution methods slightly modified previously (Kohn et al., 1981). DNA breaks were assessed in parallel in samples X-irradiated with 300 rad as positive controls.

### Results

The clonogenic inhibitory effect of DDATHF is shown in Figure 1. Dose-dependent inhibition was seen 14 days after 24 h drug exposure. The $IC_{50}$ was approximately 0.25 $\mu$M. Continuous exposure to 1–50 nM DDATHF for 48, 72, 96 or 192 h caused concentration- and time-dependent inhibition of clonogenicity in sets receiving 5, 10, 25 or 50 nM DDATHF (Figure 2). The concentration of 1 nM was not active even after 192 h exposure. The cytotoxicity of all other concentrations tested clearly increased with the exposure time.

**Figure 1** Inhibition of clonogenicity of SW626 cells growing in RPMI-1640 (containing 2.2 $\mu$M folic acid) supplemented with 10% dialysed fetal bovine serum by treatment with DDATHF for 24 h. Clonogenic potential of exponentially growing untreated control cells ranged between 85 and 90% of the cells plated, which was normalised to 100%. Data are representative of at least three independent experiments; each point is the mean of three experiments; bar, standard error of the mean.

**Figure 2** Effect of duration of treatment on SW626 cells growing in RPMI-1640 (containing 2.2 μM folic acid) supplemented with 10% dialysed fetal bovine serum. The cells were treated with 1, 5, 10, 25 and 50 nM DDATHF for 48, 72, 96 or 192 h and the colonies were stained and counted on the 14th day after seeding. Points are mean of six independent replicates; bar, standard error of the mean. □, 1 nM; ●, 5 nM; ■, 10 nM; ○, 25 nM; ▲, 50 nM.

The drug has been reported to inhibit GAR transformylase, a major regulatory enzyme in *de novo* purine biosynthesis. This causes a lack of inosinate, one of the main precursors of purines. Addition of 100 μM hypoxanthine simultaneously with 0.5 μM DDATHF reversed the inhibitory effect of the drug-induced clonogenicity (Figure 3). A lower concentration only partially reversed DDATHF cytotoxicity. This indicates that the SW626 cells have a very large need for purines when their synthesis is blocked by DDATHF. Analysis of size of colony shows that even at a high concentra-

**Figure 3** Effect of simultaneous hypoxanthine treatment on 0.5 μM DDATHF-induced inhibition of clonogenicity of SW626 cells growing in RPMI-1640 (containing 2.2 μM folic acid) supplemented with 10% dialysed fetal bovine serum. Cells were treated with 0.1, 1, 10 and 100 μM hypoxanthine with DDATHF for 24 h and colonies were allowed to develop for 14 days. Column, mean of six replicates; bar, standard error of the mean. Clonogenicity of control cells was normalised to 100%. ■, 0.5 μM DDATHF treatment for 24 h; ▨, 100 μM hypoxathine treatment for 24 h; ▩, 0.1 μM hypoxanthine together with 0.5 μM DDATHF treatment for 24 h; ▦, 1 μM hypoxanthine together wth 0.5 μM DDATHF treatment for 24 h; ▤, 10 μM hypoxanthine together with 0.5 μM DDATHF treatment for 24 h; □, 100 μM hypoxanthine together with 0.5 μM DDATHF treatment for 24 h.

**Figure 4** Cell cycle phase perturbation analysis by flow cytometry. **a**, Monoparametric (DNA) cell cycle analysis (1A–11A) and biparametric anti-BrdU immunofluorescence/DNA analysis (1B–11B). Cells treated with 0.5 μM DDATHF were analysed during 24 h of drug treatment (2A,B–5A,B) or after drug washout and followed up to 72 h (6A,b–11A,B) in drug-free medium. At specific points, cells were pulse labelled with 30 μM BrdU for 30 min, harvested, fixed in 70% ethanol and stained with anti-BrdU antibody as described in detail in Materials and methods. T, drug treatment time; R, recovery time from drug treatment. **b**, Percentages of S-phase cells (■) in the cell population and level of DNA synthesis (▲) (mean anti-BrdU green fluorescence level) of 0.5 μM DDATHF-treated cells during drug treatment (0–24 h) and after drug washout (28–96 h). Percentage of S-phase cells (□) and level of DNA synthesis (△) of exponentially growing control cells are also shown. This experiment was performed on SW626 cells growing in RPMI-1640 (containing 2.2 μM folic acid) supplemented with 10% dialysed fetal bovine serum.

tion of hypoxanthine (100 μM) the colonies were smaller than controls, suggesting that adding hypoxanthine to the medium is sufficient for colony formation but is not enough to restore the normal growth rate of SW626 in the 2 weeks after treatment (data not shown).

The drug-induced cell cycle perturbations and the level of DNA synthesis are shown in Figure 4a. Monoparametric DNA analysis (shown in the upper panels marked 1A–11A), indicated that 0.5 μM DDATHF treatment for 24 h decreased the proportion of cells in G2/M phases after an accumulation in S-middle phase of the cell cycle, up to 72 h of recovery in drug-free medium. DNA synthesis, as evaluated by uptake of 30 μM BrdU at specific points during treatment and recovery times, was established by biparametric BrdU/DNA flow cytometric analysis as shown in Figure 4(1B–11B). Between 4 h (6B) and 24 h (9B) recovery time in drug-free medium, DNA synthesis (BrdU level) progressively dropped to maximum inhibition at 24 h recovery (9B). Between 48 and 72 h recovery, the DNA synthesis rate became similar to exponentially growing untreated control cells (1B). This effect is graphically represented in Figure 4b.

The mechanism by which DDATHF-induced inhibition of purine synthesis caused its cytotoxicity is unknown. Since the inhibition of DNA synthesis appears transient and is restored completely 48 h after DDATHF washout, the cytotoxicity may not be directly related to the inhibition of DNA synthesis.

The inhibition of thymidine synthesis by MTX or CB3717 was associated with the formation of DNA breaks, and the inhibitor of protein synthesis, cycloheximide, prevented these DNA breaks, also reducing the cytotoxicity of the drug (Lorico *et al.*, 1988). Therefore, it was of interest to verify whether another antifolate that blocks purine biosynthesis without affecting thymidine synthesis also caused DNA damage and if cycloheximide could modify these DNA breaks and drug-induced cytotoxicity. As shown in Figure 5, DDATHF-induced DNA breaks were not detectable even after 48 h drug exposure, whereas under similar experimental conditions MTX caused a significant number of DNA breaks. Cycloheximide, 2.5 and 5 μM (inhibiting protein synthesis by 50% and 90% respectively in 10 min, data not shown), did not reverse the action of the drug after simultaneous application for 24 h. As shown in Figure 6, cycloheximide at the highest dose tested, 5 μM for 24 h, did not affect clonogenicity, indicating that inhibition of *de novo* protein synthesis is not involved in drug-induced cytotoxicity.

In order to assess how the folic acid content of the medium modified the clonogenic inhibitory effect of the drug, we performed different experiments using cells conditioned to grow in RPMI-1640 medium supplemented with 10% dialysed FBS, without or with different concentrations of folic acid. Figure 7 shows that the expression of mFBP assessed by using MOV18 antibody increased significantly in SW626 cells growing in the absence of folic acid. As shown in Figure 8, 0.5 μM DDATHF treatment for 24 h in total folic acid (2.2 μM) produced more than 60% inhibition of clonogenicity. In cells growing in 10–20 times less folic acid (0.22–0.11 μM), 5 nM DDATHF caused a similar level of inhibition.

**Figure 5** DNA single-strand break profile of DDATHF- and MTX-treated SW626 cells growing in RPMI-1640 (containing 2.2 μM folic acid) supplemented with 10% dialysed fetal bovine serum. ■, control cells; ○, 10 μM DDATHF treatment for 24 h and 8 h drug washout; △, 10 μM DDATHF treatment for 24 h; ▲, 10 μM DDATHF treatment for 48 h; ●, 10 μM methotrexate treatment for 24 h and 8 h drug washout; □, 300 rad X-ray-induced DNA breaks.

**Figure 6** Effect of inhibition of protein synthesis for 24 h on 0.5 μM DDATHF-induced inhibition of clonogenicity of SW626 cells growing in RPMI-1640 (containing 2.2 μM folic acid) supplemented with 10% dialysed fetal bovine serum. Cells were simultaneously treated with 2.5 (▩) or 5 (□) μM, cycloheximide and DDATHF for 24 h. ▨, 5 μM cycloheximide treatment; ■, 0.5 μM DDATHF treatment. Column, mean of six replicates; bar, standard error of the mean. Clonogenicity of control cells was normalised to 100%.

**Figure 7** Flow cytometric evaluation of the MFBP expression in SW626 cells growing in RPMI-1640 supplemented with 10% dialysed FBS with 2.2 μM (– – –) or without (······) folic acid.

**Figure 8** Effect of folic acid in the medium on DDATHF-induced inhibition of clonogenicity. The cells were treated for 24 h with 1, 5, 10, 25, 50, 100, 500 and 1000 nM DDATHF in medium containing 2.27 (□), 0.22 (●), 0.11 (■) or 0.00 (O) μM folic acid and colonies were allowed to develop for 14 days. Clonogenic potential of control cells was normalised to 100%.

When cells growing in folic acid-free medium were tested, 5 nM DDATHF completely inhibited their clonogenic potential.

Co-administration of folinic acid *in vivo* completely reversed the drug-induced systemic toxic manifestations in experimental animals (Grindey *et al.*, 1992). To study the modulation of the cytotoxic effect of DDATHF by folinic acid, we incubated the cells with different DDATHF concentrations for 24 h. Folinic acid 10 μM was added either simultaneously during the drug treatment or 0, 24, 48 and 72 h after DDATHF washout. Folinic acid was present throughout the experiment up to harvest time. Folinic acid alone did not inhibit clonogenicity of cells even at 10 μM. Concentrations of 0.25, 0.5 and 1 μM DDATHF inhibited clonogenicity by 30, 55 and 85% respectively (Figure 9). Folinic acid 10 μM added simultaneously with the drug or immediately after drug washout completely reversed the cytotoxic activity. However, when folinic acid was added after 48 h or 72 h, DDATHF still had cytotoxic activity similar to when it was used alone. A concentration of folinic acid as low as 0.1 μM was sufficient to rescue the cytotoxicity of DDATHF when given simultaneously. When folinic acid was given 24 h after DDATHF a reversal of cytotoxicity was obtained at 0.5 and 1 μM, but to a much lesser extent. As shown in Figure 10 folinic acid effect reached a plateau at 1 μM.

In order to assess the minimum time necessary for folinic

**Figure 9** Modulation of the cytotoxic potential of DDATHF by 10 μM folinic acid on SW626 cells growing in RPMI-1640 (containing 2.2 μM folic acid) supplemented with 10% dialysed fetal bovine serum. Cells were treated for 24 h with DDATHF (□) or folinic acid simultaneously with DDATHF (■), immediately after drug washout (O) and 24 h (●), 48 h (Δ) or 72 h (▲) after drug washout and analysed 14 days later. Folinic acid was present in the medium up to harvest time. Clonogenic potential of control cells was normalised to 100%.

**Figure 10** Modulation of the cytotoxic potential of 24 h treatment of 0.5 μM DDATHF by different concentrations of folinic acid on SW626 cells growing in RPMI-1640 (containing 2.2 μM folic acid) supplemented with 10% dialysed fetal bovine serum. Cells were treated with folinic acid simultaneously with DDATHF (■), immediately after drug washout (O), or 24 h (●), 48 h (Δ) or 72 h (▲) after drug washout and analysed 14 days later. Folinic acid 10 μM for 24 h did not influence the cell clonogenicity. Clonogenic potential of control cells was normalised to 100%.

**Figure 11** Modulation of the cytotoxic potential of 0.5 μM DDATHF by exposure to 1 μM folinic acid for 2, 4, 6 or 24 h on SW626 cells growing in RPMI-1640 (containing 2.2 μM folinic acid) supplemented with 10% dialysed fetal bovine serum. Cells were treated with folinic acid simultaneously with DDATHF (A), immediately after drug washout (B), and 24 h (C), 48 h (D) or 72 h (E) after drug washout and analysed 14 days later. Folinic acid for 24 h even at a concentration of 10 μM did not influence clonogenicity of the cells. Clonogenic potential of control cells was normalised to 100%. Folinic acid treatment times were 2 (■), 4 (▧), 6 (▨) and 24 h (□). ▤, 0.5 μM DDATHF treatment for 24 h.

acid to exert its modulatory effect, cells were treated for 24 h with 0.5 μM DDATHF and folinic acid at concentrations of 1, 5 or 10 μM was added for 2, 4, 6 or 24 h simultaneously with the drug or 0, 24, 48 and 72 h after DDATHF washout. Figure 11 summarises the results in 1 μM folinic acid pulse treatment since the results with 1, 5 and 10 μM folinic acid were essentially the same. Given simultaneously with DDATHF (columns marked A), folinic acid incubation for as little as 2 h completely reversed the anti-tumour potential of DDATHF. When administered immediately after 24 h DDATHF treatment and left for at least 6 h, folinic acid markedly inhibited the anti-tumour effect of drug (columns marked B). But 24 h exposure of folinic acid was essential to reverse the cytotoxic potential of DDATHF when given 24 h after DDATHF washout. Folinic acid added to the cells 48 or 72 h after drug washout for any period of time (2–24 h) did not greatly influence the anti-tumour effect of DDATHF.

All these experiments strongly suggest that during the late post-treatment periods a short or long pulse of folinic acid is equally ineffective in reducing the cytotoxic potential of DDATHF.

### Discussion

The results presented confirm that the mode of action of the antifolate DDATHF is distinct from other antifolates (Jansen *et al.*, 1991). DDATHF cytotoxicity has been related to the drug's ability to inhibit purine biosynthesis. However, the mechanism of cytotoxicity has still to be fully elucidated. In SW626 cells exposed to DDATHF the inhibition of DNA synthesis, consequent to the inhibition of *de novo* synthesis of purines, only becomes evident after a few hours and does not last long. This transient inhibition of DNA synthesis slows the progression of cells towards S-phase, but does not explain the cytotoxicity.

For other antifolates such as methotrexate (MTX), it has been proposed that cytotoxicity is due to the formation of DNA breaks, presumably caused by uracil misincorporation into DNA and/or activation of endonucleolytic enzymes (Li & Kaminskas, 1984; Lorico *et al.*, 1988). Since MTX inhibits both thymidine and purine synthesis, it has been suggested that the DNA fragmentation is triggered by the block of DNA synthesis. However, since the addition of thymidine abolishes the drug-induced DNA breakage and cytotoxicity (Lorico *et al.*, 1988), the effects may be due to thymidine deprivation. This is further supported by the fact that inhibitors of thymidylate synthase such as CB3717, which do not affect purine biosynthesis but only thymidine synthesis, also cause DNA breakage (Lorico *et al.*, 1988). The inhibitor of protein synthesis cycloheximide also inhibited MTX- or CB 3717-induced DNA breaks and cytotoxicity, suggesting that a neosynthesised protein was implicated in the mechanism of induction of DNA damage and cell death.

It was therefore of interest to study whether DDATHF, which to our knowledge is the only antifolate acting as a pure purine synthesis inhibitor, also induced DNA breakage and whether the inhibition of protein synthesis reduced the DNA damage and cytotoxicity. Our studies indicate that DDATHF cytotoxicity is not mediated by DNA breaks and that inhibition of protein synthesis does not modify the drug's activity. DNA damage caused by the previously investigated antifolates is thus probably mainly due to thymidine deprivation.

Although the mechanism of cell killing of DDATHF is related to inhibition of purine biosynthesis, as hypoxanthine blocked the effect, the drug-induced cell cycle perturbations appeared moderate and reversible, thus excluding the possibility that cytotoxicity is the consequence of prolonged blockage of DNA biosynthesis. The decrease in intracellular levels of either ATP or GTP might be the biochemical mechanism responsible for DDATHF cytotoxicity (Beardsley *et al.*, 1989; Kwok & Tattersall, 1992; Pizzorno *et al.*, 1991b).

DDATHF has high affinity for membrane folate-binding proteins (mFBP) (Antony, 1992), suggesting that differences in the expression of these proteins in neoplastic and normal tissues might be exploited to achieve drug selectivity towards some neoplasms (Jansen *et al.*, 1989, 1991). For example, in ovarian cancers mFBP, recognised by MOV18 and MOV19, have been shown to be overexpressed (Miotti *et al.*, 1987). Preclinical animal studies have indicated that folic acid strongly reduces the toxicity of DDATHF without markedly inhibiting the anti-tumour activity (Alati *et al.*, 1992; Grindey *et al.*, 1992). The concentrations of folic acid may be important in the expression of mFBP and these effects may not be the same in tumour and normal tissues.

In order to investigate the importance of the folic acid concentration we have compared the cytotoxicity of DDATHF in medium containing 2.2 μM folic acid and, after appropriate adaptation, lower concentrations. In folic acid-free medium the expression of mFBP, determined with the antibody MOV18 (Campbell *et al.*, 1991), was substantially greater in SW626 cells (Figure 7), consistent with previous reports on the regulation of mFBP expression (Antony, 1992). DDATHF sensitivity dramatically increased between 2.2 μM and 0.22 μM folic acid, with about two logs of difference in the $IC_{50}$ values. We do not know what is the explanation for this increased sensitivity. The expression of mFBP was in fact only marginally increased when cells were grown in medium containing 0.22 μM folic acid compared with cells grown in medium containing 2.2 μM folic acid (data not shown), thus suggesting that the change in drug sensitivity is not due to an induction of mFBP. The concentration of 2.2 μM, normally present in the culture medium, is approximately 100 times the physiological values of folates in human plasma, which are mainly present in the form of 5-methyltetrahydrofolate. Even with a folic acid-rich diet this concentration cannot be achieved *in vivo*. At lower concentrations of folic acid, from 0 to 0.22 μM, the differences in DDATHF cytotoxicity were smaller, indicating that in a physiological range the concentrations of folic acid only weakly influenced the inhibition of DDATHF cytotoxicity against these human ovarian cancer cells. When folic acid concentrations were below 0.22 μM a marked cytotoxicity of DDATHF at concentrations of 5–10 nM was observed. These concentrations of DDATHF can be achieved and maintained in plasma of patients receiving tolerable DDATHF dose (D.R. Newell, personal communication). This may be in line with *in vivo* data showing that folic acid dramatically reduced the toxicity but only marginally affected the anti-tumour activity in mice (G.B. Grindey, personal communication) and provide a further experimental basis to investigate the combination of folic acid and DDATHF in clinical use.

Another potentially clinically relevant aspect is whether, and to what extent, the cytotoxic effects against tumour cells are antagonised by folinic acid, normally used as an antidote for high-dose MTX and under investigation as a modulator of DDATHF toxicity (Pizzorno *et al.*, 1990; Sessa *et al.*, 1992).

In this study folinic acid completely antagonised DDATHF cytotoxicity when given simultaneously with DDATHF. The antagonism might be due to inhibition of intracellular transport, possibly by competition for the reduced folate carrier mechanism, or for mFBP, although folinic acid has been reported to have a low affinity for this high-affinity carrier. When the simultaneous treatment lasted only 2 h and DDATHF treatment was continued for a further 22 h after drug washout without any more folinic acid added, the antagonism persisted, possibly because the transport mechanism was saturated by folinic acid.

Alternatively, folinic acid may inhibit DDATHF polyglutamylation, which increases both intracellular drug retention and inhibitory potency on GAR transformylase. This is supported by previous findings in CCRF-CEM cells that folinic acid can inhibit polyglutamylation of DDATHF when given simultaneously with the drug. The same study, however, showed that if folinic acid was given 4 h after DDATHF there was no significant changes in the cellular content of the polyglutamated forms. Therefore although the antagonism observed on giving DDATHF and folinic acid simultaneously may be partly due to inhibition of polyglutamylation, it appears unlikely that this mechanism explains the antagonism observed when folinic acid is given after 24 h exposure to DDATHF. In this case it appears more likely that the inhibition of GAR transformylase by DDATHF is abolished by competition of the coenzyme, 10-methyltetrahydrofolate, rapidly formed from folinic acid (Jansen *et al.*, 1991).

The reversal of the inhibition would result in rapid restoration of purine synthesis before the purine pools drop below a threshold level and for long enough for toxicity to occur. If the interval between DDATHF and folinic acid is longer (e.g. 48 or 72 h after DDATHF treatment) purine deprivation below that threshold will probably last long enough to trigger the mechanisms of cytotoxicity (still not known, as discussed above), and addition of folinic acid can no longer save the already damaged cells.

Folinic acid is currently used in some protocols to antagonise the toxicity of DDATHF. The data of the present study suggest that there is the risk that folinic acid also blocks the anti-tumour effects. In our experimental conditions folinic acid given 24 h after DDATHF did not significantly reduce DDATHF's cytotoxic effect. Although it is difficult to extrapolate the data obtained on a cell line growing *in vitro* to the clinical situation, it seems reasonable to suggest that the interval between DDATHF and folinic acid must be of several days to avoid a reduction in anti-tumour activity, considering that human solid tumours grow much more slowly than SW626 cells.

The contributions of the Italian Association for Cancer Research, Milan, Italy; Fondazione Angelo e Angela Valenti, Milan Italy and Eli Lilly Company Bruxelles, Benelux, are gratefully acknowledged.

### References

ALATI, T., SHIH, C., POHLAND, R.C., LANTZ, R.J. & GRINDEY, G.B. (1992). Evaluation of the mechanism(s) of inhibition of the toxicity, but not the antitumor activity of Lometrexol (DDATHF) by folic acid. *Proc. Am. Assoc. Cancer Res.*, **33**, 407.

ANTONY, A.C. (1992). The biological chemistry of folate receptors. *Blood*, **79**, 2807–2820.

BALDWIN, S.W., TSE, A., GOSSETT, L.S., TAYLOR, E.C., ROSOWSKY, A., SHIH, C. & MORAN, R.G. (1991). Structural features of 5,10-dideaza-5,6,7,8-tetrahydrofolate that determine inhibition of mammalian glycinamide ribonucleotide formyltransferase. *Biochemistry*, **30**, 1997–2006.

BEARDSLEY, G.P., MOROSON, B.A., TAYLOR, E.C. & MORAN, R.G. (1989). A new folate antimetabolite, 5,10-dideaza-5,6,7,8-tetrahydrofolate is a potent inhibitor of *de novo* purine synthesis. *J. Biol. Chem.*, **264**, 328–333.

CAMPBELL, I.G., JONES, T.A., FOULKES, W.D. & TROWSDALE, J. (1991). Folate-binding protein is a marker for ovarian cancer. *Cancer Res.*, **51**, 5329–5338.

ERBA, E., SEN, S., LORICO, A. & D'INCALCI, M. (1992). Potentiation of etoposide cytotoxicity against a human ovarian cancer cell line by pretreatment with non-toxic concentrations of methotrexate or aphidicolin. *Eur. J. Cancer*, **28**, 66–71.

GRINDEY, G.B., ALATI, T., LANTZ, R., POHLAND, R. & SHIH, C. (1992). Role of dietary folic acid in blocking the toxicity but not the antitumour activity of lometrexol (DDATHF). *Ann. Oncol.*, **3** (Suppl 1), 113.

JANSEN, G., KATHMANN, I., RADEMAKER, B.C., BRAAKHUIS, B.J.M., WESTERHOF, G.R., RIJKSEN, G. & SCHORNAGEL, J.H. (1989). Expression of a folate binding protein in L1210 cells grown in low folate medium. *Cancer Res.*, **49**, 1959–1963.

JANSEN, G., WESTERHOF, G.R., KATHMANN, I., RIJKSEN, G. & SCHORNAGEL, J.H. (1991). Growth-inhibitory effects of 5,10-dideazatetrahydrofolic acid on variant murine L1210 and human CCRF-CEM leukemia cells with different membrane-transport characteristics for (anti)folate compounds. *Cancer Chemother. Pharmacol.*, **28**, 115–117.

KANE, M.A., ELWOOD, P.C., PORTILLO, R.M., ANTONY, A.C., NAJFELD, V., FINLEY, A., WAXMAN, S. & KOLHOUSE, J.F. (1988). Influence on immunoreactive folate-binding proteins of extracellular folate concentration in cultured human cells. *J. Clin. Invest.*, **81**, 1398–1406.

KOHN, K.W., EWIG, R.A.G., ERICKSON, L.C. & ZWELLING, L.A. (1981). Measurements of strand breaks and cross-links by alkaline elution. In *DNA Repair: A Laboratory Manual of Research Techniques*. Friedberg, E.C. & Hanawalt, P.C. (eds). pp. 379–401. Marcel Dekker: New York.

KRISHAN, A. & FREI III, E. (1976). Effect of adriamycin on the cell cycle traverse and kinetics of cultured human lymphoblasts. *Cancer Res.*, **36**, 143–150.

KWOK, J.B.J. & TATTERSALL, M.H.N. (1992). DNA fragmentation, dATP pool elevation and potentiation of antifolate cytotoxicity in L1210 cells by hypoxanthine. *Br. J. Cancer*, **65**, 503–508.

LI, J.C. & KAMINSKAS, E. (1984). Accumulation of DNA strand breaks and methotrexate cytotoxicity. *Proc. Natl Acad. Sci. USA*, **81**, 5694–5698.

LORICO, A., TOFFOLI, G., BOIOCCHI, M., ERBA, E., BROGGINI, M., RAPPA, G. & D'INCALCI, M. (1988). Accumulation of DNA strand breaks in cells exposed to methotrexate or $N^{10}$-propargyi-5,8-dideazafolic acid. *Cancer Res.*, **48**, 2036–2041.

MIOTTI, S., CANEVARI, S., MENARD, S., MEZZANZANICA, D., PORRO, G., PUPA, S.M., REGAZZONI, M., TAGLIABUE, E. & COLNACHI, M.I. (1987). Characterization of human ovarian carcinoma-associated antigens defined by novel monoclonal antibodies with tumor-restricted specificity. *Int. J. Cancer*, **39**, 297–303.

MORAN, R.G., TAYLOR, E.C. & BEARDSLEY, G.P. (1985). 5,10-Dideaza-5,6,7,8-tetrahydrofolic acid (DATHF), a potent antifolate inhibitory to de novo purine synthesis. *Proc. Am. Assoc. Cancer Res.*, **26**, 231.

MUGGIA, F., MARTIN, T., RAY, M., LEICHMAN, C.G., GRUNBERG, S. & GILL, I. (1990). Phase I study of weekly 5,10-dideazatetrahydrofolate (LY 264618, DDATHF-B). *Proc. Soc. Clin. Oncol.*, **9**, 74.

PIZZORNO, G., CASHMORE, A.R., MOROSON, B.A. & BEARDSLEY, G.P. (1990). Leucovorin (LV): A 'rescue' agent for 6 (R) 5,10-dideazatetrahydrofolic acid (DDATHF). *Proc. Am. Assoc. Cancer Res.*, **31**, 339.

PIZZORNO, G., SOKOLOSKI, J.A., CASHMORE, A.R., MOROSON, B.A., CROSS, A.D. & BEARDSLEY, G.P. (1991*a*). Intracellular metabolism of 5,10-dideazatetrahydrofolic acid in human leukemia cell lines. *Mol. Pharmacol.*, **39**, 85–89.

PIZZORNO, G., MOROSON, B.A., CASHMORE, A.R. & BEARDSLEY, G.P. (1991*b*). (6R)-5,10-Dideaza-5,6,7,8-tetrahydrofolic acid effects on nucleotide metabolism in CCRF-CEM human T-lymphoblast leukemia cells. *Cancer Res.*, **51**, 2291.

PIZZORNO, G., CASHMORE, A.R., MOROSON, B.A., CROSS, A.D., SMITH, A.K., MARLING-CASON, M. & others (1993). 5-10-Dideazatetrahydrofolic acid (DDATHF) transport in CCRF-CEM and MA104 cell lines. *J. Biol. Chem.*, **268**, 1017–1023.

RAY, M., MUGGIA, F., LEICHMAN, C.G., LEICHMAN, L., MORAN, R., DYKE, R. & 3 others (1992). Extended phase I study of weekly 5,10-dideaza-tetrahydrofolate (Lometrexol, DDATHF-B) in patients with advanced colorectal carcinoma. *Ann. Oncol.*, **3** (suppl. 1), 137.

SEN, S., ERBA, E. & D'INCALCI, M. (1990). Synchronisation of cancer cell lines of human origin using methotrexate. *Cytometry*, **11**, 595–602.

SESSA, C., GUMBRELL, L., HATTY, S., KERN, H. & CAVALLI, F. (1990). Phase I study of 5,10-dideazatetrahydrofolic acid (Ly264618; DDATHF) given daily for 3 consecutive days. *Ann. Oncol.*, **1** (Suppl.), P5:20.

SESSA, C., ERBA, E., SEN, S., DAMIA, G., CAVALLI, F., SCHMITT, H. & D'INCALCI, M. (1992). Folinic acid modulation of 5,10-dideazatetrahydrofolic acid (DDATHF, Lometrexol) cytotoxicity. *Proc. Am. Assoc. Cancer Res.*, **33**, 414.

TAYLOR, E.C., HAMBY, J.M., SHIH, C., GRINDEY, G.B., RINZEL, S.M. & SCHORNAGEL, J.H. (1989). Synthesis and antitumor activity of 5-deaza-5,6,7,8-tetrahydrofolic acid and its $N^{10}$-substituted analogues. *J. Med. Chem.*, **32**, 1517–1522.

WESTERHOF, G.R., JANSEN, G., VAN EMMERIK, N., KATHMANN, I., RIJKSEN, G., JACKMAN, A.L. & others (1991). Membrane transport of natural folates and antifolate compounds in murine L1210 leukemia cells: role of carrier- and receptor-mediated transport systems. *Cancer Res.*, **51**, 5507–5513.